# Supplementary material for: KDEL Receptor Trafficking to the Plasma Membrane Is Regulated by ACBD3 and Rab4A-GTP
Source: Cells. 2023 Apr 4;12(7):1079. doi: 10.3390/cells12071079 (PMC10093020; doi:10.3390/cells12071079)
Supplement: Supplementary file 1 [file cells-12-01079-s001.zip › cells-2127152-sm.pdf]

cells

Article

KDEL Receptor Trafficking To The Plasma Membrane Is Regulated by ACBD3 and Rab4A-GTP

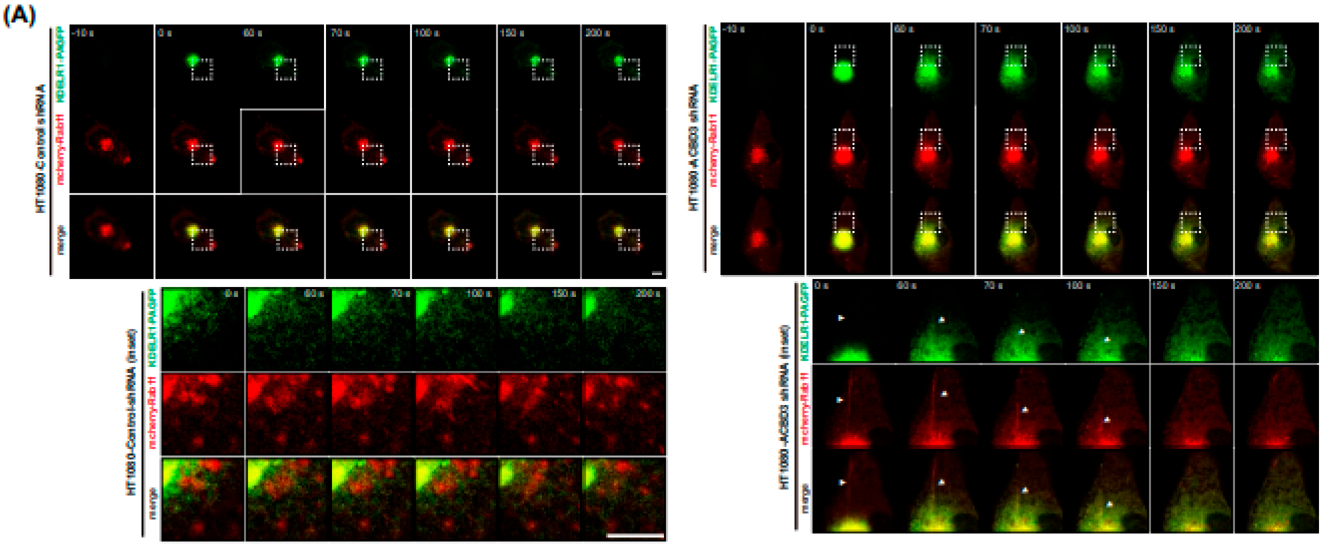

Figure S1. ACBD3 depletion results in increased trafficking of KDEL receptor to the PM, which is not mediated by Rab11A-dependent tubular carriers at the Golgi. (A-D) To examine the post-Golgi trafficking itineraries of KDEL receptor to the PM, WT or ACBD3-depleted HT1080 cells were co-transfected with photoactivatable KDELR1-PA-GFP and mCherry-Rab11A plasmids for 18 hours. The KDELR1-PA-GFP in the Golgi were then activated by selecting an ROI of mCherry-Rab11A perinuclear region for intense 405 nm laser irradiation and the transport out of the Golgi are monitored by live cell imaging acquired every 5 seconds for 5 min. Imaging sequences prior to photoactivation (-10 seconds), immediately after photoactivation (0 second) and the indicated time points following photoactivation are presented here. Magnified regions of interest (indicated by white boxes) from WT and ACBD3-KO cells at the indicated time points shows Golgi-derived tubules which are highlighted by white arrowheads. Scale bars = 5 µm.

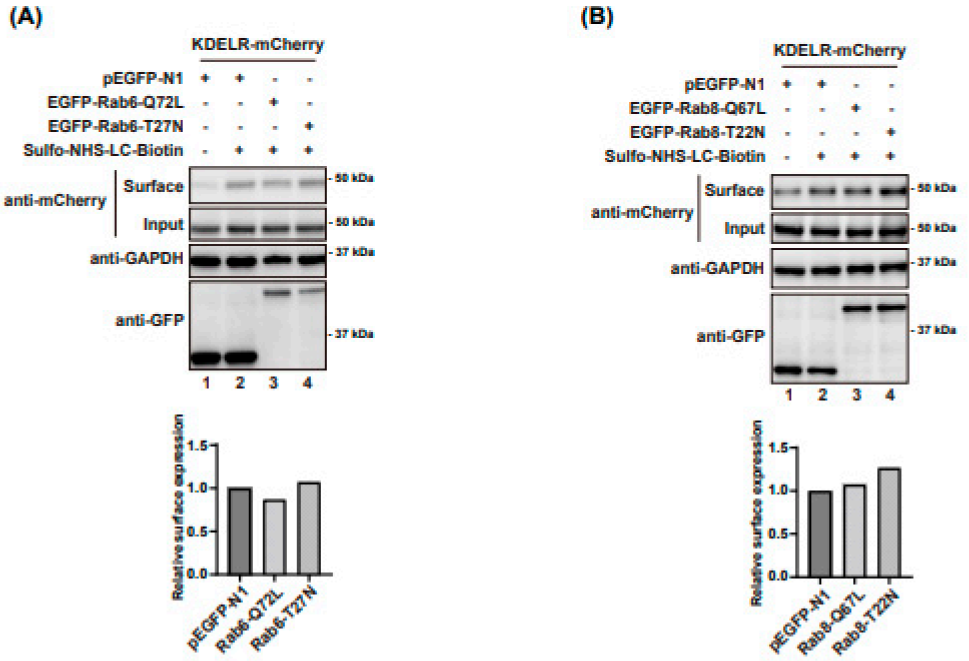

Figure S2. Rab6A and Rab8A play no role in cell surface expression of KDEL-R1. (A-B) HT1080 cells were transfected with KDEL-R1-mCherry and indicated Rab plasmids for 18 hours, followed by cell surface biotinylation. Biotinylated proteins were isolated by streptavidin-agarose and subjected to western blot analysis using the indicated antibodies. The normalized cell surface level of KDEL-R-mCherry using densitometric analysis is shown at the bottom. Statistical analysis was performed using one-way ANOVA with a Tukey's post-hoc test (mean±SD; n.s., not significant)
